# Genome-Wide Analysis of bZIP Transcription Factor Family and Its Expression in Graft Healing of Soapberry (*Sapindus mukorossi* Gaertn.)

**DOI:** 10.3390/ijms26104862

**Published:** 2025-05-19

**Authors:** Na Chen, Lixian Wang, Jing Zhong, Liming Jia, Zhong Chen

**Affiliations:** State Key Laboratory for Efficient Production of Forest Resources, Key Laboratory of Silviculture and Conservation of the Ministry of Education, National Energy R&D Center for Non-Food Biomass, Ministry of Education of Engineering Research Centre for Forest and Grassland Carbon Sequestration, College of Forestry, Beijing Forestry University, Beijing 100083, China; chenna1670441146@163.com (N.C.); wlx2018@bjfu.edu.cn (L.W.); zj5115271@163.com (J.Z.)

**Keywords:** soapberry, *bZIP* gene family, evolutionary analysis, expression pattern, grafting

## Abstract

The Basic Leucine Zipper (bZIP) transcription factors play a vital role in plant responses to abiotic stress. Despite being studied in various plant species, the function of the *bZIP* gene family in Soapberry (*Sapindus mukorossi* Gaertn.), a significant tree species for forestry biomass energy, remains unclear. In this study, we conducted a genome-wide analysis of the *bZIP* gene family in Soapberry, based on the observation that bZIP transcription factors were enriched in the transcriptome data of Soapberry-grafted stem segments, as revealed by both GO and KEGG analyses. For the first time, we identified 31 *SmbZIP*s and provided detailed information regarding their physicochemical characteristics, gene structures, protein motifs, phylogenetic relationships, *cis-*regulatory elements (CREs), and predicted transcriptional regulatory networks. According to our prediction of the *SmbZIP*-mediated regulatory network and CREs in the promoter region, *SmbZIP*s may be associated with plant growth and development as well as responses to mechanical wounding stress. By integrating RT-qPCR and RNA-seq analyses, we determined that the expression patterns of *SmbZIP*s were specific to the graft-healing stages and locations. In conclusion, our study elucidates the potential role of the *bZIP* gene family in responding to plant wounding stress and facilitating graft healing, thereby providing valuable insights for future functional genomics studies of Soapberry.

## 1. Introduction

Plants encounter various abiotic stressors, including high salinity, drought, low temperatures, and mechanical wounding, during their growth and development. Numerous molecular, cellular, physiological, and biochemical responses have been established to counteract these pressures [1]. Among these responses, transcription factors play a crucial role in the transmission of stimulus signals and the activation of genes involved in stress responses [2]. Recent studies have identified several families of transcription factors, including Basic Leucine Zipper (bZIP), NAM-ATAF-CUC (NAC), APETALA2/Ethylene Responsive Factor (AP2/ERF), and MY-ELOB-LASTOSIS (MYB), that are associated with the abiotic stress response [3,4,5,6]. In particular, bZIP transcription factors are essential for enhancing plants’ resilience to abiotic stressors.

The bZIP family is one of the largest and most diverse transcription factor (TF) families, named after its conserved bZIP domain. The structural characteristics that define the bZIP domain, comprising approximately 60–80 amino acid residues, include the following: (1) a C-terminal region characterized by a leucine zipper motif containing the L-x6-L-x6-L core sequence, which consists of various repeated leucine or other hydrophobic amino acids [7]; (2) an N-terminal region that is basic and contains a nuclear localization signal along with an N-x7-R/K motif that binds to specific DNA *cis-*elements [8]. Interactions between the hydrophobic surfaces in this region frequently lead to the formation of homo- or hetero-dimers in the form of α-helices. bZIP proteins preferentially bind to DNA sequences featuring an ACGT core, which includes the A-box, C-box, and G-box [9]. Furthermore, bZIP transcription factors have been extensively utilized in genetic engineering to enhance plant stress resistance across various species, including barley (*Hordeum vulgare*), soybean (*Glycine max*), tribulus clover (*Medicago truncatula*), and Arabidopsis (*Arabidopsis thaliana*). The response of *AtbZIP29* to osmotic stress in Arabidopsis has been documented [10]. Overexpression of *MtbZIP2* and *MtbZIP26* has been shown to improve the salt tolerance of plants [11]. Additionally, increased expression of *GmbZIP102* [12] and *HvABI5* [13] has been demonstrated to aid plants in adapting to drought stress. The number of *bZIP* genes varies among species, ranging from 53 in Chinese chestnut (*Castanea mollissima*) [14], 55 in grape (*Vitis vinifera*) [15], 69 in tomato (*Solanum lycopersicum*) [16], 92 in barley [17], 114 in apple *(Malus domestica*) [18], 125 in maize (*Zea mays*) [19], to over 200 in rapeseed (*Brassica napus*) [20]. However, the identification and functional characterization of *bZIP* genes in Soapberry (*Sapindus mukorossi*) remain unreported.

Soapberry is recognized as an excellent tree species for biomass energy, possessing significant economic, ecological, and cultural value [21]. The elite cultivated varieties of Soapberry utilize grafting techniques to accelerate breeding and maintain stable, desirable traits. However, grafting involves severing vascular bundles [22], which subjects the plant to abiotic stressors, including mechanical damage and water stress during the initial healing phases [23]. Transcriptome analysis of grafted Soapberry stem segments reveals that the bZIP transcription factor *SmABI5* is significantly up-regulated in the early stages of graft healing. We hypothesize that bZIP members act as critical regulators of abiotic stress in plants, facilitating Soapberry graft healing by responding to water stress and mechanical injury early in the healing process. Therefore, the availability of the complete Soapberry genome provides an excellent opportunity to assess the *bZIP* genes in Soapberry and explore their potential roles in abiotic stress adaptation.

In this study, we identified a total of 31 *SmbZIP* genes based on the Soapberry genome. We provided a comprehensive characterization of these genes, including their physicochemical properties, chromosomal locations, gene structures, motif compositions, evolutionary relationships, *cis*-regulatory elements (CREs), collinearity analyses, and transcription factor (TF) regulatory networks. Our findings may clarify the potential roles of the *bZIP* gene family in Soapberry’s response to mechanical damage stress. Furthermore, transcriptome data from various healing stages of scions and rootstocks after grafting indicate that *SmbZIP* genes exhibit tissue-specific expression across different regions and times in the grafted stem segments of Soapberry. Overall, our findings enhance the understanding of the evolution and future roles of the bZIP family while establishing a foundation for the effective breeding, comprehensive development, and utilization of superior Soapberry cultivars.

## 2. Results

### 2.1. Examining Differentially Expressed Genes in Soapberry Associated with Graft Union

**Figure 1:**
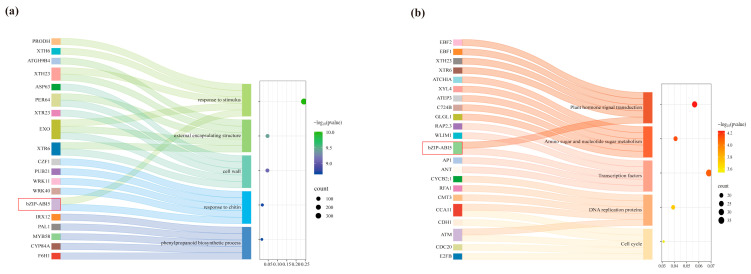
(**a**) Analysis of the DEGs in the S2 vs. S1 comparison group GO functional annotation and (**b**) KEGG pathway enrichment. Red box shows labeled ABI5 transcription factor.

We employed RNA-sequencing technology to analyze the gene expression profiles of Soapberry at different healing stages after grafting. The correlation and cluster analyses between biological replicate samples showed the dependability of the transcriptome data. The initial phase following grafting is a critical time for wound healing [24]. In the study of genes linked to abiotic stress reactions, identifying the important genes during the wound response stage is significant. The S2 vs. S1 comparison group has 2593 differentially expressed genes (DEGs) based on an analysis of RNA-seq data. These DEGs’ GO (gene ontology) functional analysis revealed that they were enriched in processes like cell wall, stimulus–response, and phenylpropanoid biosynthesis (Figure 1a). In the meantime, transcription factors and plant hormone signal transduction were among the KEGG (Kyoto Encyclopedia of Genes and Genomes) pathways that these DEGs enhanced (Figure 1b). Notably elevated in both S1 and S2 was ABA-insensitive 5/DPBF1 (ABI5), which was found in both investigations. We hypothesize that the particular expression of the ABI5 transcription factor in the *bZIP* gene family contributes to the initial wound healing rate in plants.

### 2.2. Identification and Characterization of SmbZIPs in Soapberry

**Figure 2:**
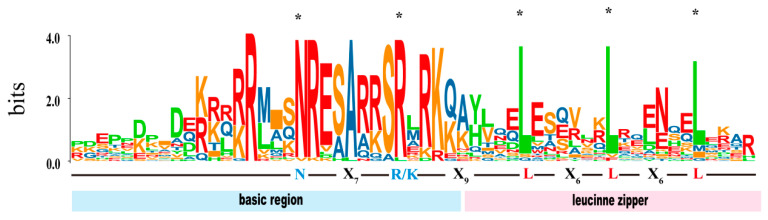
An illustration of the multiple-sequence alignment of the Soapberry’s bZIP family domains. The overall height of the letter stack represents the sequence conservation at each point. The height of each letter in the letter stack shows the relative frequency of the corresponding amino acid at a given position. Black asterisks indicate asparagine (N) and basic (R/K) residues with exact spacing.

The whole-genomic protein sequences of Soapberries were searched using the bZIP domain (PF00170) and the basic region leucine zipper domain (PF07716) using the Hidden Markov Model (HMM) and BLASTp methods [9]. We further improved the integrity and dependability of the *SmbZIP* candidates based on the modifications in the amino acid sequences [25]. Following the elimination of unnecessary and redundant genes, 31 *SmbZIP*s were found in the Soapberry genome and given names based on where they were found on the chromosome (Table S1). A multiple-sequence alignment of the *SmbZIP* amino acid sequences was carried out to investigate the conserved domains of the discovered *SmbZIP* proteins. All 31 *SmbZIP* proteins have a conserved domain composed of a basic DNA-binding region and a nearby leucine zipper structure, according to the data (Figure 2). The ZIP domain is made up of heptad repeats of leucine (L) or other hydrophobic amino acids, whereas the basic DNA-binding region has an invariant N-X7-R/K pattern [26]. The number of repeat sequences varies as a result of the occasional substitution of isoleucine, methionine, and more for the highly conserved leucine residues [9]. Our findings are in line with earlier research on poplar (*Populus przewalskii*) and Arabidopsis [9,27]. This means that the *SmbZIP* members vetted by Soapberry are trustworthy. After that, we analyzed their physicochemical characteristics.

The average length of the *SmbZIP* gene family’s coding sequence is 949 base pairs (bp), with a range of 348 to 2091 bp. The average length of the bZIP protein sequences is 315 amino acids, with a range of 115 to 696 amino acids. The majority of the encoded SmbZIP proteins are hydrophilic (Aliphatic Index < 100), acidic (Theoretical pI < 7), and unstable (Instability Index > 40) proteins, with an average molecular mass of 35.23 kDa. Meanwhile, the findings of *SmbZIP*s’ subcellular localization show that they are all found in the nucleus. This finding is in agreement with the conventional knowledge of transcription factors, which holds that the nucleus is where transcription factors regulate gene transcription [28]. These results support earlier observations in other plant species [14,29] and offer a theoretical foundation for more SmbZIP protein purification, activity, and functional research.

### 2.3. Chromosomal Location and Analysis of SmbZIPs

**Figure 3:**
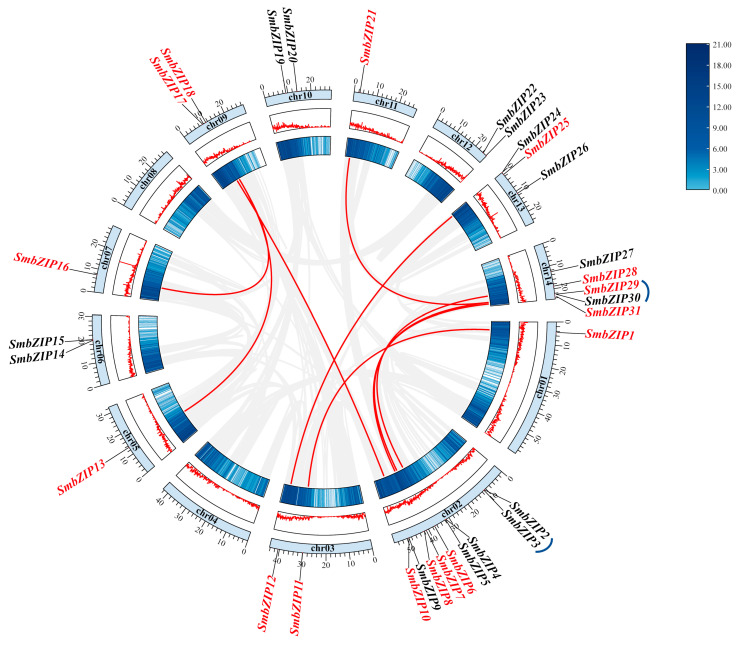
Chromosomal localization and collinearity analysis of *SmbZIP*s. Inside each chromosome are the corresponding numbers. The inner circle’s lines and heatmap show the chromosomes’ gene density. The gray curves indicate the co-localization of genetic loci in the Soapberry genome. Red highlights the collinear gene pairings and indicates collinear interactions between *SmbZIP* genes. Red text indicates co-linear *SmbZIP* genes. Blue lines represent tandem duplications for *SmbZIP* genes.

To investigate the chromosomal locations of *bZIP* genes in the Soapberry genome, we performed in silico mapping analysis of the genetic loci. In accordance with our research, the 14 Soapberry chromosomes have an unequal distribution of the 31 *SmbZIP*s (Figure 3). In particular, chr02 received nine *SmbZIP*s, whereas chr04 and chr08 received none. Different gene duplication modes, including whole-genome, tandem, and proximal duplication, are responsible for the growth of gene families [30]. Every duplication method is thought to be a significant factor in the evolution of species and makes an individual contribution to evolution [31]. To uncover the gene duplication events in Soapberry, researchers used the MCScanX approach to conduct an analysis [32]. Two pairs of tandem gene duplications and nine pairs of chromosomal segment duplications were found. Based on our findings, the primary mechanism behind the evolution of *SmbZIP*s is chromosomal segment duplication events, which is in line with the published studies on *bZIP*s in Chinese chestnuts [14]. All of these processes can be regarded as ways for species to diversify and adapt to unfavorable external circumstances [33].

This study used the One-Step MCScanX-Super Fast function in the TBtools program to calculate the non-synonymous substitution rate (Ka), synonymous substitution rate (Ks), and Ka/Ks ratio of conjugate gene pairs to understand the selection pressure impacting the evolution of *SmbZIP*s (Table S2). To ascertain whether a selection pressure is operating on a protein-coding gene, one can use the Ka/Ks ratio of two protein-coding genes [34]. As a result, this ratio was chosen to represent the species’ evolutionary selection. The research results showed that most of the computed Ka/Ks ratios were less than 1, suggesting that strong purifying selection had an important impact on the evolution of the *SmbZIP* family. For certain synonymous gene pairs, the ratio could not be calculated (the value was displayed as “NaN”). This could be a result of the majority of these genes’ mutation sites being synonymous, which indicates an enormous sequence divergence and a lengthy evolutionary distance [35].

### 2.4. Phylogenetic Relationship and Collinearity Analysis of SmbZIPs

**Figure 4:**
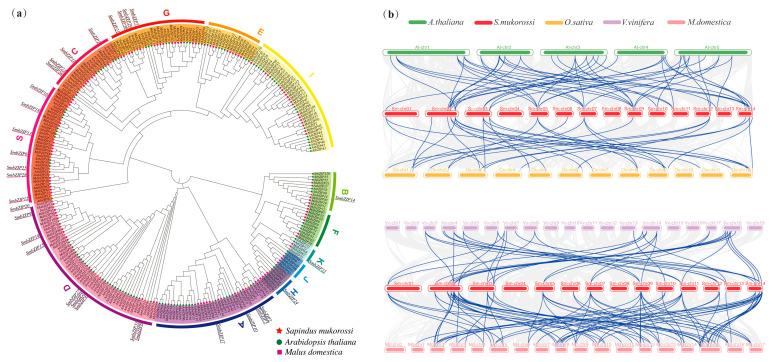
*SmbZIP* phylogenetic connections. (**a**) The maximum-likelihood (ML) option with 1000 replicates was used to generate the phylogenetic tree of the *bZIP* gene family and the phylogenetic tree of the bZIP proteins from Soapberry (*Sapindus mukorossi*), Arabidopsis (*Arabidopsis thaliana*), and apple (*Malus domestica*). The protein sequences of the apple, Arabidopsis, and Soapberry are shown by the medium-purple squares, dark-green circles, and red stars, respectively. (**b**) *bZIP* gene homology analysis between 4 representative plants and Soapberry. The steel-blue lines draw attention to the collinear *bZIP* gene pairs, while the gray lines in the backdrop show the collinear blocks in the genomes of Soapberries and other plants. ‘*A. thaliana*’, ‘*O. sativa*’, ‘*V. vinifera*’, and ‘*M. domestica*’ are prefixes that indicate Arabidopsis, rice, grape, and apple, respectively.

To explore the evolutionary relationships of the *bZIP* gene family among different plant species, we merged the protein sequences of *bZIP*s from Soapberry, Arabidopsis, and apple. We then used MEGA11 to create a maximum-likelihood (ML) phylogenetic tree (Figure 4a). The 13 groups of Arabidopsis’s bZIP family members are named after their important genes (e.g., A for ABF/AREB/ABI5, C for CPRF2-like, G for GBF, H for HY5), their protein size (B for large, S for small), or alphabetically [9]. The phylogenetic analysis reveals that the 272 bZIP proteins from the three species may be separated into 12 subgroups (A–K, S) based on earlier research on Arabidopsis and apples [9,18]. Each of the 12 groups has a different number of members. Group D (9) and Group S (6) are both of the larger groups. Groups A, C, and G all have four members each, while Groups E, F, I, and J do not have *SmbZIP*s. Although C, E, G, I, and S are all members of the same subcluster, their distributions differ significantly, indicating that they may have shared an ancestor but that the distribution of these genes in the species has been impacted by evolutionary diversity. To demonstrate the homologous relationships of *bZIP* genes in various species, using four representative species—three dicotyledonous plants (Arabidopsis, apple, and grape) and one monocotyledonous plant (rice)—a collinearity map was created (Figure 4b). The findings revealed a tight genetic connection between *SmbZIP*s and *MdbZIP*s, with the former displaying more homology (Table S3).

Furthermore, Soapberry has a higher collinearity with the genomes of dicotyledonous plants than with those of monocotyledonous plants, indicating that most of these orthologous pairings formed subsequent to the divergence of monocotyledons and dicotyledons. Among them, a close evolutionary link between the chosen species is indicated by the 13 *SmbZIP*s that exhibit homologous relationships with the 4 example species (Table S3). Plus, these genes may have conserved evolutionary activities and have greatly influenced the development of the bZIP family. The comparison of the *SmbZIP* family with those of other species provides a research reference for examining the genetic relationships and gene functions among species.

### 2.5. Gene Structure, Conserved Motif Analysis of SmbZIPs

**Figure 5:**
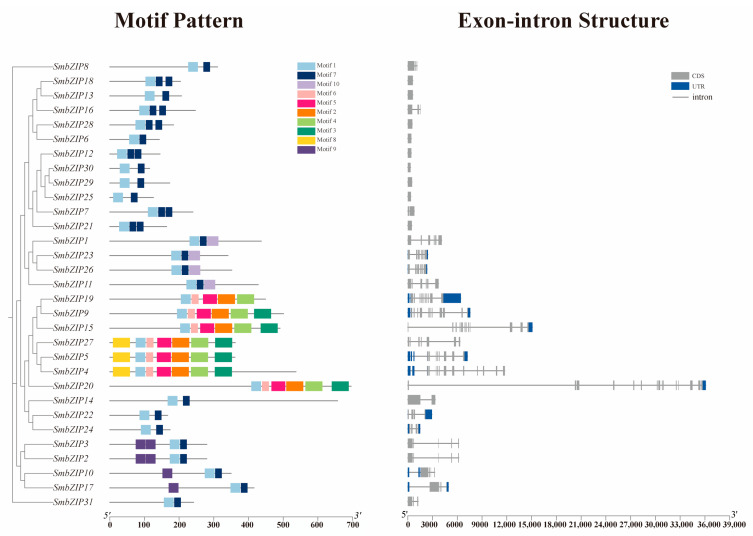
Conserved motifs and gene structure of the *SmbZIP* gene family. Rectangles of different hues are used to pinpoint the conserved motifs. The gene structure consists of untranslated regions (UTR, steel-blue rectangle), exons (CDS, gray rectangle), and introns (gray line). The lengths of various genes and proteins are compared using the scale at the bottom.

Determining the connection between the *bZIP* gene family’s evolution and functional differentiation requires the use of gene structure analysis. To examine the structural characteristics of 31 *SmbZIP*s, 10 conserved motif patterns, designated motif 1–10 (Figure 5), were found using MEME motif analysis. Every member possesses a minimum of two motifs. Of these, Group D (*SmbZIP4*, *SmbZIP5*, and *SmbZIP27*) has the highest number of motifs (7) and the same motif order. Although different motifs appeared in different subgroups, the existence of similar motif structures in bZIP members of the same subgroup indicates that these proteins are relatively conserved. This outcome further supports the validity of the *SmbZIP* gene family classification. Moreover, motif 1 is present in all *SmbZIP* members, which contains the highly conserved N-x7-R/K motif that binds to particular DNA sequences and is the primary feature of the bZIP conserved domain [7]. Using the Soapberry GFF annotation file, the arrangement of exons and introns in the open reading frames of *SmbZIP*s was examined in order to better understand the evolution of *bZIP* genes in Soapberry. Group S is home to the majority of intron-less genes, which is consistent with studies on Arabidopsis [7,9]. This suggests that there is a strong correlation between gene structure and the evolutionary relationships between members of the gene family (Figure 5).

### 2.6. Prediction Analysis of SmbZIP-Mediated Regulatory Network

**Figure 6:**
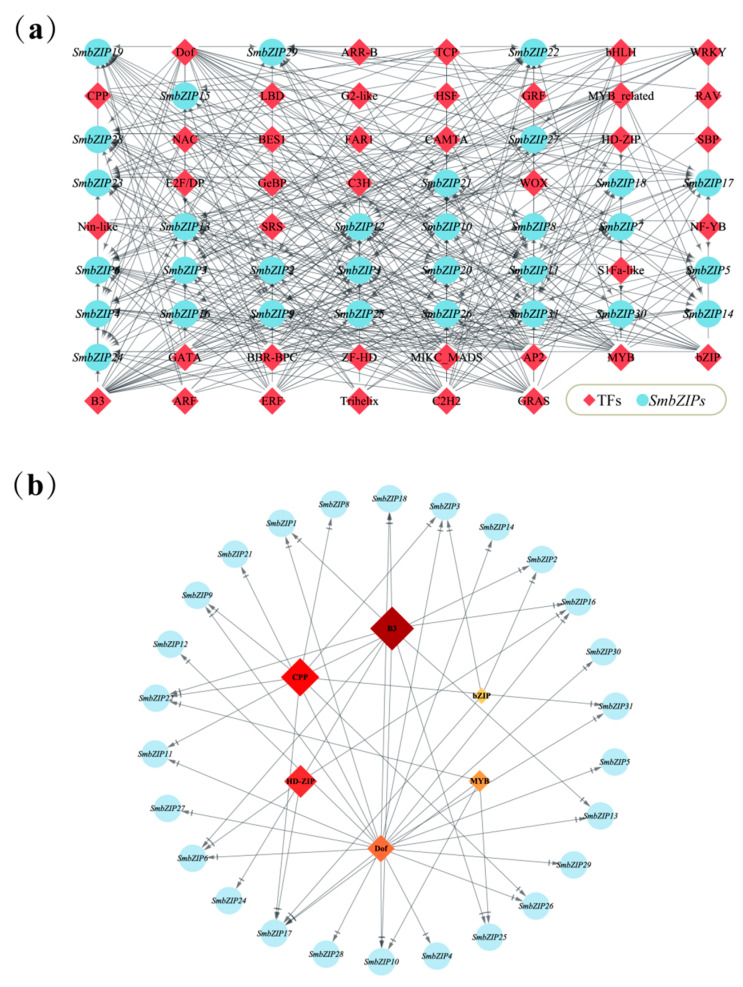
*SmbZIP*s’ anticipated TFs regulatory network. (**a**) *SmbZIP*s are represented by the light-blue circular nodes, while TFs are represented by the orange diamond nodes. The top eight highly enriched and targeted *SmbZIP*s are displayed in (**b**). The enrichment increases with color darkness.

Multiple TFs regulate gene expression [30]. To forecast the transcriptional regulatory linkages mediated by *SmbZIP*s, the promoter sequences were examined in the PlantRegMap database. A total of 447 TFs from 39 TF families were found to be putative transcription factors for *SmbZIP*s (Figure 6a, Table S4). According to the prediction results, the ERF family’s TFs are the most prevalent (72), followed by the MYB family’s (51). In contrast, there is only one component in each of the ARR-B, FAR1, GRAS, GRF, NF-YB, RAV, and S1Fa-like families. Conversely, *SmbZIP6* has the most regulatory factors (100) of any *SmbZIP*, next to *SmbZIP1* and *SmbZIP28* (86). Multiple members from several TF families target the *SmbZIP*s in Group E. The BBR-BPC family’s targets are the most enriched among them (16). Members of AP2 (15) are expected to target *SmbZIP23*, B3 (15) will target *SmbZIP29*, bHLH (22) will target *SmbZIP11*, and NAC (14) will target *SmbZIP14*. Furthermore, this study discovered that grafting investigations have documented six of the top eight highly enriched gene families, which are TCP, B3, CPP, ZF-HD, HD-ZIP, Dof, MYB, and bZIP (Figure 6b). For instance, HD-ZipIII controls the vascular cambium’s xylem properties and stem cells’ organizing capabilities [36,37]. The xylem formation gene cluster is located downstream of *MYB86* [38]. Dof is activated by cell wall disruption and aids in tissue regeneration and wound healing in Arabidopsis [39]. By selectively binding to cell-cycle regulators and genes involved in cell wall regeneration, *AtbZIP29* facilitates tissue regeneration. Due to these results, they might be crucial in controlling the plant graft-healing process [10]. The aforementioned findings indicate that the *SmbZIP*-mediated regulatory network may be connected to the networks of plant growth and development as well as the response to stress caused by mechanical injury.

### 2.7. Analysis of Cis-Regulatory Elements of SmbZIPs

**Figure 7:**
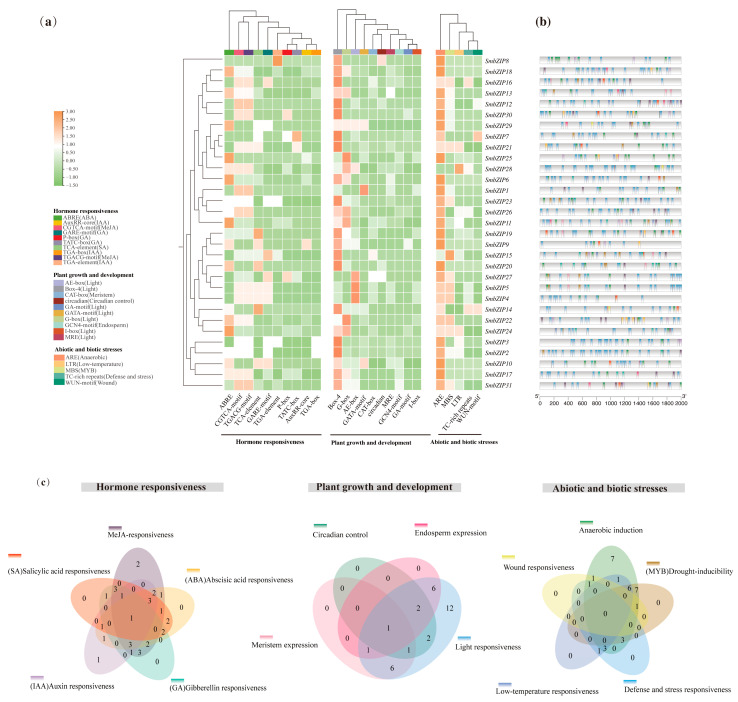
CREs in *SmbZIP*s. (**a**) The CRE heatmap for the 2000 bp area upstream of the *SmbZIP*s transcription start point. (**b**) The distribution of these elements within the promoter sequences is displayed. Different colors represent different types of CREs, as in (**c**). (**c**) A Venn diagram showing different CREs, with each number denoting how many *SmbZIP*s have that element.

Gene expression depends on CREs. TFs work with the matching CREs found in the promoter regions of stress-responsive genes to encourage the buildup of functional proteins, thereby increasing plant stress tolerance [40]. The sequences in the 2000 bp region upstream of the transcription start sites of 31 *SmbZIP* genes were evaluated in the PlantCARE database for the purpose of gaining a better understanding of the potential roles of *SmbZIP*s in plant growth and development, plant hormones, and stress responses. Figure 7a shows the typical CREs out of the 756 CREs that were anticipated (Table S5). *SmbZIP9* in Group D had the fewest distributed CREs (9) among them, but *SmbZIP18* and *SmbZIP28* in Group S had the most (37) (Figure 7b). They were separated into three main groups based on their functions (Figure 7c): (i) Auxin responsiveness (IAA, 9.9%), salicylic acid responsiveness (SA, 9.4%), gibberellin responsiveness (GA, 14.6%), abscisic acid responsiveness (ABA, 25.5%), and MeJA-responsiveness (40.6%) are among the hormone responses (192). Among them, ABRE, CGTCA-motif, and TGACG-motif, which are involved in ABA response and MeJA response, were more prominent (Figure 7a). Further, 10 typical hormone-responsive elements were found in the promoter region of *SmbZIP28*, showing that it may react to particular hormones more quickly and robustly. (ii) Associated with plant development and growth (429), such as meristem expression (2.6%), endosperm expression (2.3%), circadian control (1.9%), and light response (93.2%). Elements associated with the light response act extensively on the promoter regions of *SmbZIP*s. Plant *bZIP*s typically preferentially bind to palindromic or pseudo-palindromic *cis-*regulatory elements with an ACGT core, such as the G-box, A-box, and ABRE, in accordance with pertinent research [7,9]. Furthermore, 10 growth and development-related representative elements were discovered in the promoter region of *SmbZIP11*, indicating that it may have an important regulatory function in the process of plant growth and development. (iii) Associated with biotic and abiotic stressors (123), such as wound responsiveness (3.2%), defense and stress response (6.5%), low-temperature responsiveness (10.6%), anaerobic induction (60.2%), and drought inducibility (MYB, 19.5%). While the drought-inducible element (MBS) and wound-responsive element (WUN motif) mainly appeared in Group D of *SmbZIP*s, the ARE element, which is implicated in anaerobic induction, appears in nearly all *SmbZIP*s (Figure 7a). The findings demonstrated that distinct *SmbZIP*s have variable numbers and compositions of CREs in their promoter regions. Numerous CREs linked to hormones, plant growth and development, and stress responses may control the functional expression of *bZIP* genes in Soapberry.

### 2.8. SmbZIP Gene Expression in Scion and Rootstock Tissues

**Figure 8:**
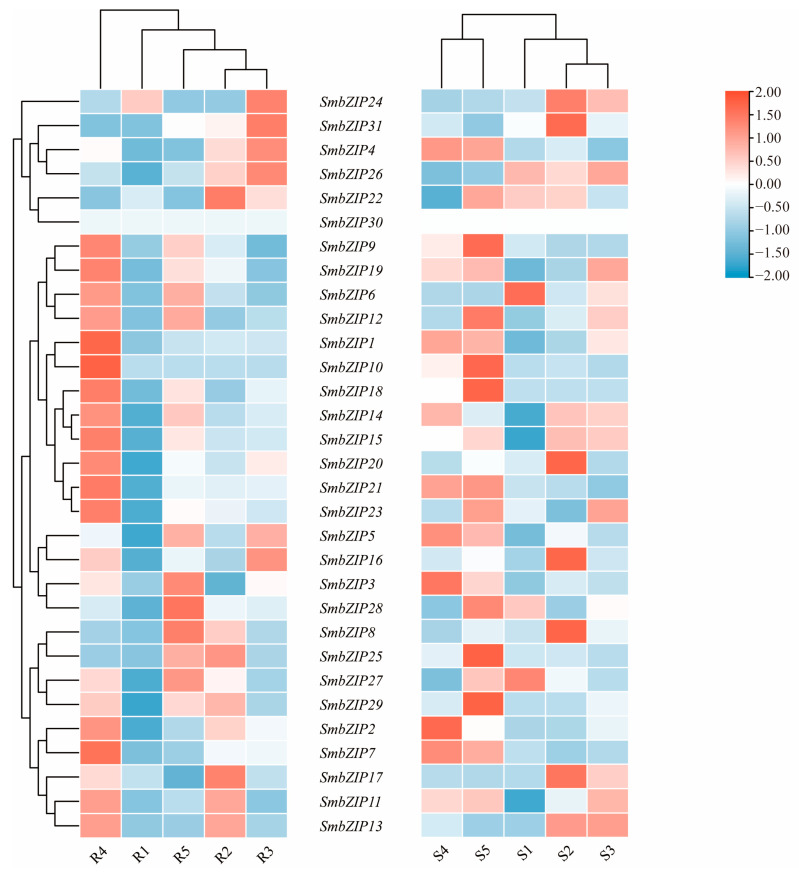
Expression levels of *SmbZIP* genes in Soapberry at five separate graft-healing stages. Here, S stands for the scion, and R for the rootstock. Along with the clustering-related expression levels, the heatmap’s red, white, and blue hues stand for high, medium, and low expression levels, respectively.

To validate the expression patterns of *SmbZIP* genes related to the response to mechanical injury, we used the unpublished transcriptome data of Soapberry grafted stem segments to calculate the fragments per kilobase of transcript per million mapped reads (FPKM). High, medium, and low expression levels are denoted by red, white, and blue in the heatmap, respectively. The pertinent expression levels were grouped (Figure 8). During the S1 stage, *SmbZIP6*, *SmbZIP26*, and *SmbZIP28* were substantially expressed and activated in Group S, and the scions’ expression levels were greater than the rootstocks’. During the S2 and R2 stages, members of Group D (*SmbZIP8*, *SmbZIP17*, *SmbZIP20*, and *SmbZIP31*) displayed up-regulated expression, which was followed by a decrease in expression levels. This suggests that the presence of scions corresponds to the reactions in the grafted rootstocks and that *SmbZIP* genes have functional biases towards both rootstocks and scions, meaning they are specific to direction and time. During the rootstock–scion connection stage, members of Group K (*SmbZIP22*) and Group H (*SmbZIP24*) exhibited comparatively high expression levels. Interestingly, most *SmbZIP*s had high expression levels throughout the R4 and S5 stages, but the great majority of members had low expression levels during the R1 stage. The activation of genes linked to vascular reconnection may be connected to the elevated gene expression of *SmbZIP*s.

### 2.9. Analysis of qRT-PCR Gene Expression in SmbZIP Gene Famliy

**Figure 9:**
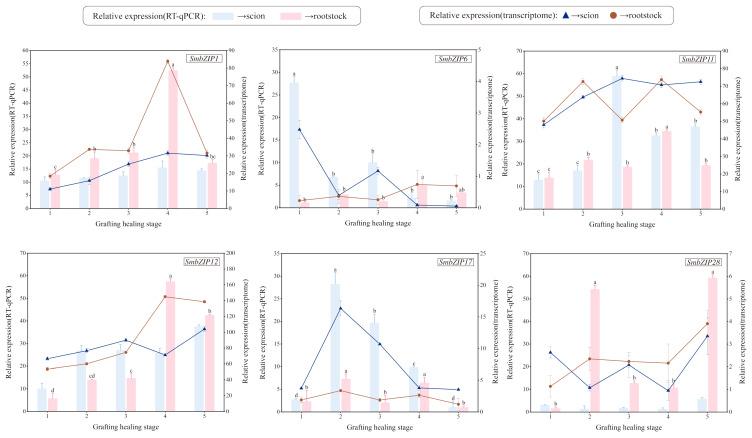
Validation of the differentially expressed results obtained by RNA-seq. RT-qPCR and RNA-seq analyses were used to ascertain the relative expression levels of 6 genes throughout the five stages of Soapberry graft healing. Different letters indicate significant differences, and the same letters represent no significant differences. The bars show the standard deviation.

To validate the RNA-Seq results, we employed quantitative real-time PCR (qRT-PCR) to measure the expression levels of six DEGs in different regions during the graft-healing process after selecting them at random. *SmbZIP6* was substantially expressed in the scion at the first stage (S1), whereas *SmbZIP28* displayed a higher expression level in the rootstock in the second stage (R2), as shown in Figure 9. Following the cell division stage and the rootstock–scion connection stage (i.e., stages 2 and 3), the expression levels of *SmbZIP11* and *SmbZIP17* declined. Additionally, the RNA-Seq results and the qRT-PCR results for *SmbZIP1* and *SmbZIP12* were essentially in agreement.

## 3. Discussion

The two primary issues currently impacting global development are the energy crisis and climate change [41]. Future energy solutions must fulfill the requirements for renewable energy and low carbon emissions [42]. A pivotal pathway for China’s energy transition involves replacing fossil fuel usage with forestry biomass energy, driven by the principle of not competing with food crops for land [43]. However, the cultivation of Soapberry, a significant source of forestry biomass energy, is hindered by a lengthy development cycle and genetic instability. Consequently, grafting techniques are essential for preserving its desirable and stable traits while reducing breeding time. In this context, a critical phase in genetic improvement through genetic engineering approaches is the identification of beneficial gene resources that enhance graft healing. Given the importance of *bZIP*s in plant responses to abiotic stress and the enrichment of *SmABI5* in both GO and KEGG analyses of the transcriptome data from grafted stem segments of Soapberry, it is logical to conduct an in-depth investigation of *bZIP*s to identify potential candidates involved in the mechanical damage response.

This study found 31 *SmbZIP*s using the genomic data from the first edition of the Soapberry genome. According to previous research, gene families typically experience tandem or segmental duplication during the evolutionary process, which results in the emergence of new gene functions and expression patterns while preserving the family’s size [44]. The number of *bZIP* genes in various species varies as well because the mutation rates of *bZIP* genes change during gene duplication [9,45]. The expansion of the *SmbZIP* gene family was primarily accomplished through chromosomal segmental duplication, according to the investigation of the collinear relationship among *SmbZIP*s. Additionally, tandem duplication is essential for the formation of abiotic stress-responsive genes [46]. It is interesting to note that Soapberry only has one set of tandem repeats of the *bZIP* genes, which is in keeping with research conducted on rice and poplar [8,27]. These findings imply that segmental duplication has contributed substantially to the *bZIP* gene family’s growth.

Multiple sequence alignments show that the conserved bZIP domains in Soapberry and Arabidopsis are identical [14,18]. Based on the bZIP family’s evolutionary research, *AtbZIP*s are divided into 13 different groups. The 31 members of Soapberry were split up into 12 subgroups (A-K, S) based on their similarities with *AtbZIP*s. Throughout development, members of Groups D and S have a prominent position, typically having a larger subclass and a comparatively high number. Interestingly, many patterns appear in particular groupings, which could be connected to particular biological processes. Only Group A, which includes the ABF/AREB subgroup, contains motif 9. Abiotic stress triggers the transcription of factors in the ABF/AREB subgroup, which are then post-transcriptionally activated in the ABA signaling pathway to start an adaptive response [47]. Motif 9 might contain *cis-*elements for genes activated by abscisic acid. During the plant’s healing phase, grafting operations may cause water stress. Water deficiency can be tolerated by transgenic Arabidopsis that overexpresses *AtABF2* and *AtAREB1* [48,49]. To show the evolutionary links between species and to find potential orthologs and paralogs, phylogenetic analysis is frequently used. Homologous genes typically maintain comparable roles and cluster into the same subgroup [50]. Consequently, we performed a functional prediction on the members of Clade A that contain the ABF/AREB subgroup (*SmbZIP2*, *SmbZIP3*, *SmbZIP10*, and *SmbZIP17*) and are more likely to be involved in controlling the wound response during the early stages of graft healing. Notably, the transcriptome data of the Soapberry grafted stem segments revealed that *SmbZIP17* is exactly the differentially expressed and up-regulated gene *SmABI5*. In addition, motif 10 is exclusive to Group C. Sugar signaling in plants has been linked to a number of bZIP transcription factors in Group C, including *bZIP9*, 10, 25, and 63 [51]. Studies have demonstrated that sugar is not only necessary for graft healing but also facilitates the growth of grafted plants [52,53]. We hypothesize that motif 10 might have particular binding sites for glycosyl synthesis or transfer and that overexpressing Group C members might also aid in Soapberry graft healing. Phylogenetically conserved components of *SmbZIP*s consistently have similar exon–intron architectures and motifs, as shown in other plants, including apples and Chinese chestnuts [14,16]. This suggests that different bZIP proteins might have similar functions. Motif 1 is the most conserved of the 10 conserved motifs found in all *SmbZIP* proteins. It has a conserved bZIP domain, which is essential to these TFs’ functional specificity [7].

We discovered that genes in Group S hardly ever include introns based on the findings of gene structure analysis. Their compact gene structure suggests their possible role in plants’ quick reaction to different abiotic stressors [54]. The number of regulatory variables for *SmbZIP6* and *SmbZIP28* in Group S is comparatively high, as expected, given the regulatory network mediated by *SmbZIP*s. In biological signal transduction pathways, transcription factors’ binding to the CREs in the promoter region is crucial for gene expression [55]. The promoter regions of *SmbZIP*s were shown to have a variety of regulatory components linked to hormone control, growth and development, and stress response. These elements included those involved in anaerobic induction, abscisic acid-responsive elements, and light-responsive elements. By specifically targeting the G-box, HY5, the bZIP transcription factor can form a dynamic activation-repression transcriptional module with PIF that reacts to temperature and light, according to research by G. Toledo-Ortiz et al. [56]. *ThbZIP1* binds to the A-box of stress-response genes in bristly tamarisk (*Tamarix hispida*), regulating the expression of downstream genes [57]. Induced by environmental stress, the ABRE containing ACGT encodes bZIP TFs (*AtABF1*, *AtABF2*, *AtABF3*, and *AtABF4*) in Arabidopsis, which plays a role in salt and drought tolerance [58]. Evidently, the co-presence of three different CREs in the promoter region of *SmbZIP*s may be strongly related to the potential regulatory influence of *SmbZIP*s on plant growth and development under abiotic stress. Additionally, we observed that *SmbZIP28* contains a number of CREs associated with plant growth and development (light responsiveness, circadian control, endosperm expression), hormone response (MeJA responsiveness, abscisic acid responsiveness, gibberellin responsiveness, Auxin responsiveness, salicylic acid responsiveness), and stress response (low-temperature responsiveness, defense and stress responsiveness). It means that *SmbZIP28* may be a critical regulator of plant growth and development in addition to being a significant regulator of plant response to mechanical damage stress.

This study investigates the transcriptional levels of *SmbZIP* genes under abiotic stress in a time- and tissue-specific manner. Despite the constraints of sampling time points, the Soapberry internodes exhibited a similar pattern. In contrast, *SmbZIP6* and *SmbZIP28* expression levels in the scion and rootstock of Group S displayed opposing patterns during the early phases of the wound response. We speculate that during the initial stage of the wound response, the gene expression level in the tissue below the incision induced by grafting approaches the high expression level in the tissue above the incision. This suggests that the presence of the scion promotes the response in the grafted rootstock. A gene’s function is often partially reflected in the degree of its expression at various time points and in different tissues [59]. The expression of *SmbZIP10* and *SmbZIP18* was exclusively elevated during the vascular reconnection stage, leading us to postulate that *SmbZIP10* and *SmbZIP18* contribute to the onset of phloem and xylem reconnection, which warrants further investigation. Rt-qPCR tests confirmed the expression pattern of each gene during graft healing and validated the accuracy of the transcriptome data from the grafted stem segments of Soapberry in this study.

Recent studies have identified certain gene expression patterns that correlate with the morphological changes occurring during the graft-healing process. Notaguchi et al. generated virus-induced gene silencing (VIGS) and overexpression lines, demonstrating that *NbGH9B* in tobacco (*Nicotiana benthamiana*) plays a significant role in cell wall repair at the grafting interface using a wedge grafting approach [60]. In tomato, CRISPR-Cas9-mediated knockdown of *SIWOX4*, followed by oblique/wedge grafting, confirmed its crucial role in vascular reconnection at the graft junction [38]. In summary, *SmbZIP11*, *SmbZIP17*, and *SmbZIP28* are important candidate genes for further investigation and detailed characterization to elucidate their roles in graft healing.

## 4. Materials and Methods

### 4.1. Plant Materials

The scion was sourced from the ‘YUANHUA’ clone, while the rootstock utilized in this study comprised two-year-old ‘YUANHUA’ seedlings grown from seeds. The ‘YUANHUA’ clone and seeds of Soapberry from Soapberry National Forest Germplasm Banks in Jianning County, Fujian Province, China (26°49′N, 116°52′E, 300 m above sea level). The upper, sunlit portion of the rootstock was selected for grafting. Grafting was performed using the cut technique, with an incision length of approximately 1.5 cm. Following grafting, the plants were maintained at a temperature of 17.5 ± 2.8 °C under a relative humidity of 77 ± 3%. The four main stages of the Soapberry graft-healing process are the wound response stage (0–12 days post-grafting, indicated as d), the cell division stage (13–19 d), the scion–rootstock connection stage (20–24 d), and the vascular reconnection stage (25–60 d), according to research our group conducted [24]. The rootstock and scion stem segments were taken at five different time intervals after grafting: 7 d, 14 d, 21 d, 30 d, and 45 d. With ‘R’ standing for the rootstock and ‘S’ for the scion, these segments were numbered R1, R2, R3, R4, and R5 for the rootstock and S1, S2, S3, S4, and S5 for the scion, respectively. The specimens were kept in liquid nitrogen as soon as they were sampled. The FinePure Plant Total RNA Extraction Kit (GENFINE BIOTECH Co., Ltd., Beijing, China) was used to extract the samples’ total RNA. The quality of the extracted RNA was next evaluated using the Bioanalyzer 2100 (Agilent, Palo Alto, CA, USA) to ensure the samples met the requirements for generating cDNA libraries. Beijing YUANYI BIOTECH Co., Ltd. (Beijing, China) was responsible for the library’s high-throughput sequencing (Illumina HiSeq X Ten, San Diego, California, USA). High-quality clean data were produced after low-quality sequences, adapter sequences, and sequences with uncertain base N were eliminated from the raw data acquired from high-throughput sequencing [61]. Using programs like HISAT2 (v2.2.1), RSeQC (v4.0.0), and String Tie (v2.2.3), the clean data were subsequently put through sequence alignment and transcript assembly with the Soapberry reference genome that our research team had previously created [62].

### 4.2. Gene Screening for Differential Expression

Each transcript’s read count was determined, and the abundance value of gene expression was measured using the FPKM (fragments per kilobase of transcript per million fragments mapped). The study of differential gene expression between samples was carried out using DESeq2 (v1.20.0) [63]. DEGs were defined as genes having a Benjamini–Hoffberg adjusted *p*-value < 0.05 and |log_2_(fold change)| > 1. R (v4.1.0) software was used to conduct the KEGG and GO enrichment analysis of the DEGs. The Bioinformatics Cloud Platform (https://www.bioinformatics.com.cn/login/, accessed on 11 August 2022) was used to visualize the results.

### 4.3. Identification of bZIP Genes in Soapberry

The National Genomics Data Center (NGDC) database (https://ngdc.cncb.ac.cn, accessed on 3 December 2024) provided the most recent annotation files for the Soapberry reference genome, which were used to build a local database. In the meantime, the entire genome’s Genome-Wide Haplotype (GWH) number is WGS069104, which is available on PRJCA019364. The Arabidopsis genome-wide information was obtained from the Arabidopsis Information Resource (TAIR) database (http://www.arabidopsis.org/, accessed on 3 December 2024) in order to identify every possible member of the *bZIP* genes in Soapberry. BLASTp (Basic Local Alignment Search Tool for proteins) was used to search the Soapberry genome using the amino acid sequences of 127 *AtbZIP* proteins as a reference. For the TBtools (v2.146) program [64], the E-value (≤1 × 10^−5^) and identity match (≥50%) were established as the cutoff points. The Hidden Markov Model (HMM) profiles of the basic region leucine zippers (PF07716) and bZIP domain (PF00170) were downloaded from the Protein Family (Pfam) database (http://pfam-legacy.xfam.org/, accessed on 3 December 2024) [65]. An E-value (≤1 × 10^−5^) was utilized as the standard domain for HMM homologous sequence searches with HMMER 3.3.1 (http://hmmer.org/download.html, accessed on 3 December 2024) [66]. To further confirm candidate protein sequences, the putative *SmbZIP*s found by the BLASTp and Hmmer searches were filtered, combined, and then added to the NCBI Conserved Domain Database (https://www.ncbi.nlm.nih.gov/cdd/, accessed on 3 December 2024). The REtrotransposed Gene EXPlorer was used to remove superfluous proteins and pseudogenes [67].

### 4.4. Analysis of Protein Physicochemical Properties and Chromosomal Distribution

The Gene Location Visualise function in TBtools (v2.146) software [60] was applied to map and identify the chromosomal locations of *SmbZIP*s based on the genomic annotation data of Soapberry. The gene loci of the *SmbZIP*s were used to rename them. To predict and assess the physicochemical properties, including the number of amino acids, molecular weight, and theoretical pI, the ExPASy Prot-Param tool (https://web.expasy.org/protparam/, accessed on 6 December 2024) was used [68]. Lastly, WoLF PSORT (https://wolfpsort.hgc.jp/, accessed on 6 December 2024) estimated the protein’s subcellular location [69].

### 4.5. Multiple Sequence Alignment and Phylogenetic Analysis

The complete genome information of the apple (*Malus domestica*), grape (*Vitis vinifera*), and rice (*Oryza sativa*) was obtained from the National Center for Biotechnology Information (NCBI) database (https://www.ncbi.nlm.nih.gov/, accessed on 3 December 2024) and the Genome Database for Rosaceae (GDR) database (https://www.rosaceae.org/, accessed on 3 December 2024) to observe the evolutionary relationship of the *bZIP* gene family. The Muscle program in MEGA11 software was used to analyze the multiple sequence alignment of *SmbZIP*s, *AtbZIP*s, and *MdbZIP*s with the default parameter settings [70]. The Jones–Taylor–Thornton model (JTT + G) was used to build a maximum-likelihood (ML) phylogenetic tree with the resulting multiple-alignment file. One thousand bootstrap replicates were utilized to assess relative branch support, pairwise genetic distance comparison was utilized to determine branch lengths, and pairwise deletion was utilized for processing missing data. For better visualization, the phylogenetic tree was beautified using the Interactive Tree Of Life (ITOL) v6 online tool (https://itol.embl.de/, accessed on 2 January 2025) [71].

### 4.6. Analysis of Conserved Motifs and Gene Structure

The Multiple Em for Motif Elicitation (MEME v5.5.7) online application (https://meme-suite.org/meme/, accessed on 11 December 2024) was used to find conserved motifs in the SmbZIP proteins [72]. The maximum number of bases was adjusted to 10 in the program options, while other settings were left at their default defaults. The GFF annotation file of the Soapberry genome provided the intron–exon distribution of the *SmbZIP* genes. Lastly, the Gene Structure View in TBtools (v2.146) was used to visualize the data [64].

### 4.7. Analysis of Collinearity

The downloaded genome sequence files and genome structure annotation information files were analyzed using the One-Step MCScanX-Super Fast and File Transformat for MicroSynteny Viewer in TBtools (v2.146) software with the goal of retrieving the collinearity information of *bZIP* genes both within and between species [64]. To see the collinearity relationships between the members of the bZIP family, all of the output files were loaded into the Advanced Circos in TBtools (v2.146) program. All duplicate gene pairs within *SmbZIP*s had their Ka and Ks values as well as their Ka/Ks values determined using the Ka/Ks Calculator in the TBtools (v2.146) software.

### 4.8. Analysis of Cis-Regulatory Elements

The Sequence Toolkit in TBtools (v2.146) software [64] was used to recover the 2000 bp sequences upstream of the start codons of each *SmbZIP* gene. Potential *cis-*regulatory elements were then found using the PlantCARE database (http://bioinformatics.psb.ugent.be/webtools/plantcare/html/, accessed on 24 December 2024) [73]. The TBtools (v2.146) software’s Gene Structure View, HeatMap, and Venn functions were used to process and visualize the findings manually.

### 4.9. Analysis of TF Regulatory Network

The possible regulatory interactions of transcription factors (TFs) in the promoter regions (2000 bp) of *SmbZIP*s were inferred using the Plant Transcription Regulatory Map (PTRM) (http://plantregmap.gao-lab.org/, accessed on 21 December 2024) [74], a regulatory prediction tool, with a threshold (*p*-value ≤ 1 × 10^−5^). Moreover, Arabidopsis was the chosen plant species. The Cytoscape software (v3.9.1) was used to show the predicted TFs as a network [75].

### 4.10. Expression Patterns of SmbZIP Genes

The transcriptome data of grafted stem segments of Soapberry were normalized using TBtools (v2.146) [64] to produce a gene expression pattern map, which was then utilized to examine the variations in *SmbZIP* gene expression between the scion and rootstock tissues.

### 4.11. Quantitative Real-Time PCR Analysis

Using Primer Premier 5.0 [76], specific primers were created and produced by RUIBO XINGKE BIOTECH Co., Ltd. (Beijing, China). Table S6 contains comprehensive primer sequence data. The Taq Pro Universal SYBR qPCR Master Mix reagent (Vazyme, Nanjing, China) was used to perform qRT-PCR analysis on the Applied Biosystems^®^ QuantStudioTM 6 Flex real-time fluorescence quantitative PCR system (Thermo Fisher Scientific, Waltham, MA, USA). To normalize the data, *SmACT* was employed as an internal reference gene [77]. Every experiment was conducted at least three times on its own. The 2^−∆∆Ct^ technique was used to determine the expression levels [78]. Variance analysis was performed using IBM SPSS (v26), and significance was examined using one-way ANOVA (and nonparametric or mixed). The associations between the expression profiles of six chosen genes identified by RT-qPCR and RNA-seq were shown using Origin (v2021).

## 5. Conclusions

This study provides the initial description of *bZIP* genes in Soapberry, focusing on their potential functions in response to plant-damaging stress. A total of 31 *SmbZIP* genes were identified, distributed unevenly across 14 chromosomes. Most *SmbZIP*s were characterized as hydrophilic, unstable, and acidic proteins. The *SmbZIP* gene family was categorized into 12 subgroups based on a phylogenetic tree derived from protein sequences, revealing both sequence conservation and functional divergence among these subgroups. Notably, insights into the potential biological roles of *SmbZIP*s in plant growth and development, as well as their involvement in mechanical injury stress responses, are elucidated through the *SmbZIP*s-mediated regulatory network, *cis*-regulatory elements, and expression patterns observed at various graft-healing stages. Particularly, members of clades A and C, especially *SmbZIP11* and *SmbZIP17*, along with *SmbZIP6/28* from Group S, may significantly influence the regulation of wound responses during graft healing. Our research initially examined the genetic relationships among *SmbZIP*s, establishing a theoretical framework for the functional investigation of key genes involved in Soapberry graft healing. This foundation will facilitate the validation of these genes’ functions and may be leveraged for future genetic engineering initiatives.

## Data Availability

The authors declare that the data supporting the findings of this study are available within the paper and its Supplementary Information files. Should any raw data files be needed in another format, they are available from the corresponding author upon reasonable request.

## Supplementary Materials

The following supporting information can be downloaded at: https://www.mdpi.com/article/10.3390/ijms26104862/s1.
